# Relationship between *Helicobacter pylori* infection and digestive tract diseases and analysis of risk factors: a cross-sectional study based on 3867 Chinese patients

**DOI:** 10.18632/aging.206065

**Published:** 2024-08-22

**Authors:** Wang Zhao, Yanzhi Han, Yizhi Xiao, Yuan Liu, Zhenling Zhang, Lijuan Liao, Jinqi Wei, Xiaofeng Li, Minzhao Gao, Jing Lu

**Affiliations:** 1 Department of Gastroenterology, The Fifth Affiliated Hospital of Sun Yat-sen University, Zhuhai 519000, China

**Keywords:** *Helicobacter pylori*, digestive tract diseases, infection rate, risk factor

## Abstract

*Helicobacter pylori* (*H. pylori*) infect nearly half of the global population, contributing to upper digestive tract diseases. This 2019 cross-sectional study included 3,867 patients undergoing esophagogastroduodenoscopy (EGD) and 2,875 undergoing both colonoscopy and EGD. Subjects were categorized into *H. pylori* positive and negative groups by rapid urease test (RUT). In addition to exploring the relationship between *H. pylori* infection and upper gastrointestinal diseases, this study further revealed that *H. pylori* infection was closely related to lower digestive tract diseases, including colorectal polyp (63.28%) and colorectal cancer (75.76%), as well as upper and lower gastrointestinal comorbidities, including chronic atrophic gastritis with colorectal polyp (79.85%), peptic ulcer with colorectal polyp (79.72%), gastric polyp with colorectal polyp (66.24%), and chronic atrophic gastritis with colorectal cancer (92.86%). Besides, a univariate logistic regression analysis was conducted to compare the differences between the two groups (including gender, nationality, marital status, smoking history, drinking history, living area, age, BMI, glycosylated hemoglobin, fasting blood glucose, total cholesterol, and triglyceride levels), the results identified marital status and age as independent risk factors for *H. pylori* infection (OR, 1.435; 95% CI, 1.042 to 1.977; OR, 1.007; 95% CI, 1.001 to 1.013). Further clarification of the correlation between the prevalence of gastrointestinal diseases and *H. pylori* infection will be important for *H. pylori* infection management strategies and the treatment and prevention of gastrointestinal diseases.

## INTRODUCTION

The Gram-negative microaerophilic bacterium *Helicobacter pylori* (*H. pylori*) is identified as a key pathogenic agent that can be transmitted from person to person, causing chronic gastritis, peptic ulcer, and gastric cancer [1–3]. Since 1994, the International Agency for Research on Cancer has categorized *H. pylori* as a Group 1 carcinogenic agent [4]. Research has shown that approximately 50% of the world’s population is infected with *H. pylori* [5], with a median infection rate of 44.2% in China (95% CI, 43.0–45.5%), representing nearly 600 million infected individuals [6]. Despite recent studies suggested a gradual decline in *H. pylori* infection rates in numerous developed nations, the prevalence continues to be alarmingly high in the majority of developing countries [5]. Therefore, understanding the risk factors for *H. pylori* infection is of utmost importance.

Numerous studies have shown that *H. pylori* infection not only affects the stomach and proximal duodenum, but also the intestinal tract on the anal side. For instance, meta-analyses have found that individuals infected with *H. pylori* have a higher prevalence of colorectal adenomas and cancers [2, 7–11]. However, the relationship between *H. pylori* and lower digestive tract (such as colorectal tumors or polyps) remains controversial, with most studies having relatively small sample sizes [12, 13]. Furthermore, no research has yet revealed the association between *H. pylori* infection and the prevalence of comorbidities in the upper and lower digestive tract.

In summary, we conducted a retrospective analysis of the database containing information on 3867 Chinese patients. The aim of this research was to identify any potential associations between various diseases of the upper and lower digestive tract and *H. pylori* infection. Additionally, we aimed to identify the risk factors associated with *H. pylori* infection, thereby facilitated the formulation of evidence-driven decision-making frameworks and targeted intervention strategies.

## RESULTS

### Social-demographic, clinical and related characteristics of the study participants

A total of 3,867 patients were included in the study, consisting of 1,885 male patients and 1,982 female patients. Based on the results of RUT, the patients were categorized into *H. pylori* infection positive and negative group. Subsequently, a univariate logistic regression analysis was conducted to compare the differences in gender, nationality, marital status, smoking history, drinking history, living area, age, BMI, glycosylated hemoglobin, fasting blood glucose, total cholesterol, and triglyceride levels between the two groups. According to analysis, a significant difference was observed in marital status between the two groups (*P* < 0.05). The infection rate of *H. pylori* in married individuals was 63.08%, which was significantly higher than that in single individuals. However, there were no significant differences in gender, nationality, smoking history, drinking history, and living area between the *H. pylori* positive and negative groups (*P* > 0.05) (Table 1). Additional, significant differences were observed in age and fasting blood glucose levels between the two groups (*P* < 0.05). In terms of age, the mean ± SD age of patients in the *H. pylori* positive group was 53.07 ± 11.55 years, while the mean ± SD age of patients in the negative group was 51.82 ± 11.82 years, there was significant statistical significance between the two groups (*P* < 0.001). In terms of fasting blood glucose, the mean ± SD glucose level of patients in the *H. pylori* positive group was 5.28 ± 1.16 mmol/L, while 5.22 ± 1.23 mmol/L in the negative group. The difference in fasting blood glucose levels between the two groups was also statistically significant (*P* < 0.045). Nonetheless, there were no significant differences in BMI, glycosylated hemoglobin, total cholesterol, and triglyceride levels between the *H. pylori* positive and negative group (*P* > 0.05) (Table 1).

**Table 1:** Social demographic, clinical and other related characteristics of study participants.

**Variables**	**Total** **(*n* = 3867)**	***H. pylori* infection (+)** **(*n* = 2419)**	***H. pylori* infection (−)** **(*n* = 1448)**	***P-*value**
Gender				0.236
Male	1885	1197 (63.50)	688 (36.50)	
Female	1982	1222 (61.65)	760 (38.35)	
Nationality				0.401
Ethnic Han	3843	2402 (62.50)	1441 (37.50)	
Ethnic minorities	24	17 (70.83)	7 (29.17)	
Marital status				0.002^**^
Married	3694	2330 (63.08)	1364 (36.92)	
Single	173	89 (51.45)	84 (48.55)	
Smoking history				0.922
Yes	774	483 (62.40)	291 (37.60)	
No	3093	1936 (62.59)	1157 (37.41)	
Drinking history				0.899
Yes	543	341 (62.80)	202 (37.20)	
No	3324	2078 (62.52)	1246 (37.48)	
District				0.105
Xiangzhou	2744	1742 (63.48)	1002 (36.52)	
Doumen	751	445 (59.25)	306 (40.75)	
Jinwan	372	232 (62.37)	140 (37.63)	
Age, years	52.60±11.66	53.07 ± 11.55	51.82 ± 11.82	0.001^**^
BMI, kg/m^2^	23.44±3.19	23.50 ± 3.23	23.33 ± 3.11	0.130
Glycosylated hemoglobin, %	5.83±0.87	5.84 ± 0.85	5.82 ± 0.90	0.117
Fasting blood-glucose, mmol/L	5.26±1.18	5.28 ± 1.16	5.22 ± 1.23	0.045^*^
Total cholesterol, mmol/L	4.99±1.04	5.00 ± 1.06	4.96 ± 1.00	0.376
Triglyceride, mmol/L	1.56±1.17	1.59 ± 1.25	1.50 ± 1.00	0.068

### Relationship between upper digestive tract diseases and *H. pylori* infection rate

All the 3867 patients in this study have undergone gastroscopy, 2419 cases (62.55%) were diagnosed to have *H. pylori* infection. Among them, the infection rates of *H. pylori* in patients with esophageal squamous cell cancer, chronic atrophic gastritis, gastric ulcer, duodenal ulcer, compound ulcer, gastric polyp, gastric MALT lymphoma and gastric cancer were 77.27%, 80.48%, 87.32%, 78.96%, 92.00%, 65.09%, 87.50% and 93.33%, respectively, and there was significant statistical difference between the infected groups and non-infected groups (*P* < 0.05) (Table 2 and Supplementary Table 1).

**Table 2:** Comparison of *H. pylori* infection rate in patients with upper digestive tract diseases.

**Disease**	***H. pylori* infection (+)** **(*n* = 2419)**	***H. pylori* infection (−)** **(*n* = 1448)**	**Total** **(*n* = 3867)**	**Infection rate**	***P-*value**
Reflux esophagitis	182	173	355	51.27%	*P* < 0.001
Esophagus cancer	34	10	44	77.27%	0.042^*^
Chronic atrophic gastritis	169	41	210	80.48%	*P* < 0.001
Gastric ulcer	62	9	71	87.32%	*P* < 0.001
Duodenal ulcer	259	69	328	78.96%	*P* < 0.001
Compound ulcer	23	2	25	92.00%	0.002^**^
Gastric polyp	783	420	1203	65.09%	0.029^*^
Gastric MALT lymphoma	14	2	16	87.50%	0.039^*^
Gastric cancer	28	2	30	93.33%	*P* < 0.001

### Relationship between lower digestive tract diseases and *H. pylori* infection rate

Among the 3867 patients included in this study, 2875 patients underwent colonoscopy either simultaneously or within three months. Among them, the infection rates of *H. pylori* in colorectal polyp and colorectal adenocarcinoma patients were 63.28% and 75.76%, respectively, and there was significant statistical difference between the infected groups and non-infected groups (*P* < 0.05) (Table 3 and Supplementary Table 2).

**Table 3:** Comparison of *H. pylori* infection rate in patients with lower digestive tract diseases.

**Disease**	***H. pylori* infection (+)** **(*n* = 1765)**	***H. pylori* infection (−)** **(*n* = 1110)**	**Total** **(*n* = 2875)**	**Infection rate**	***P-*value**
Colorectal polyp	1308	759	2067	63.28%	0.001^**^
Colorectal cancer	75	24	99	75.76%	0.003^**^
Inflammatory bowel disease	19	12	31	61.29%	0.991

### Relationship between upper and lower digestive tract comorbidities and *H. pylori* infection rate

The study included a total of 2875 patients who underwent gastroenteroscopy during the same period, we also analyzed the relationship between *H. pylori* infection and comorbidities of the upper and lower digestive tract. The *H. pylori* infection rates among patients with chronic atrophic gastritis and colorectal polyp, peptic ulcer and colorectal polyp, gastric polyp and colorectal polyp, as well as chronic atrophic gastritis and colorectal adenocarcinoma were found to be 79.85%, 79.72%, 66.24%, and 92.86% respectively, and the infection rate in the infected groups was significantly higher than that in the non-infected groups (*P* < 0 .05) (Table 4).

**Table 4:** Comparison of *H. pylori* infection rate in patients with upper and lower digestive tract comorbidities.

**Disease**	***H. pylori* infection (+)** **(*n* = 1765)**	***H. pylori* infection (−)** **(*n* = 1110)**	**Total** **(*n* = 2875)**	**Infection rate**	***P*-value**
Reflux esophagitis and Colorectal polyp	101	100	201	50.25%	0.001^**^
Esophagus cancer and Colorectal polyp	4	3	7	57.14%	*P* > 0.05
Chronic atrophic gastritis and Colorectal polyp	107	27	134	79.85%	*P* < 0.001
Peptic ulcer and Colorectal polyp	169	43	212	79.72%	*P* < 0.001
Gastric polyp and Colorectal polyp	471	240	711	66.24%	0.002^**^
Gastric cancer and Colorectal polyp	5	1	6	83.33%	*P* > 0.05
Reflux esophagitis and Colorectal cancer	8	3	11	72.73%	*P* > 0.05
Chronic atrophic gastritis and Colorectal cancer	13	1	14	92.86%	0.015^*^
Peptic ulcer and Colorectal cancer	8	1	9	88.89%	*P* > 0.05
Gastric polyp and Colorectal cancer	27	8	35	77.14%	*P* > 0.05

### Independent risk factors related to *H. pylori* infection

**Figure 1:**
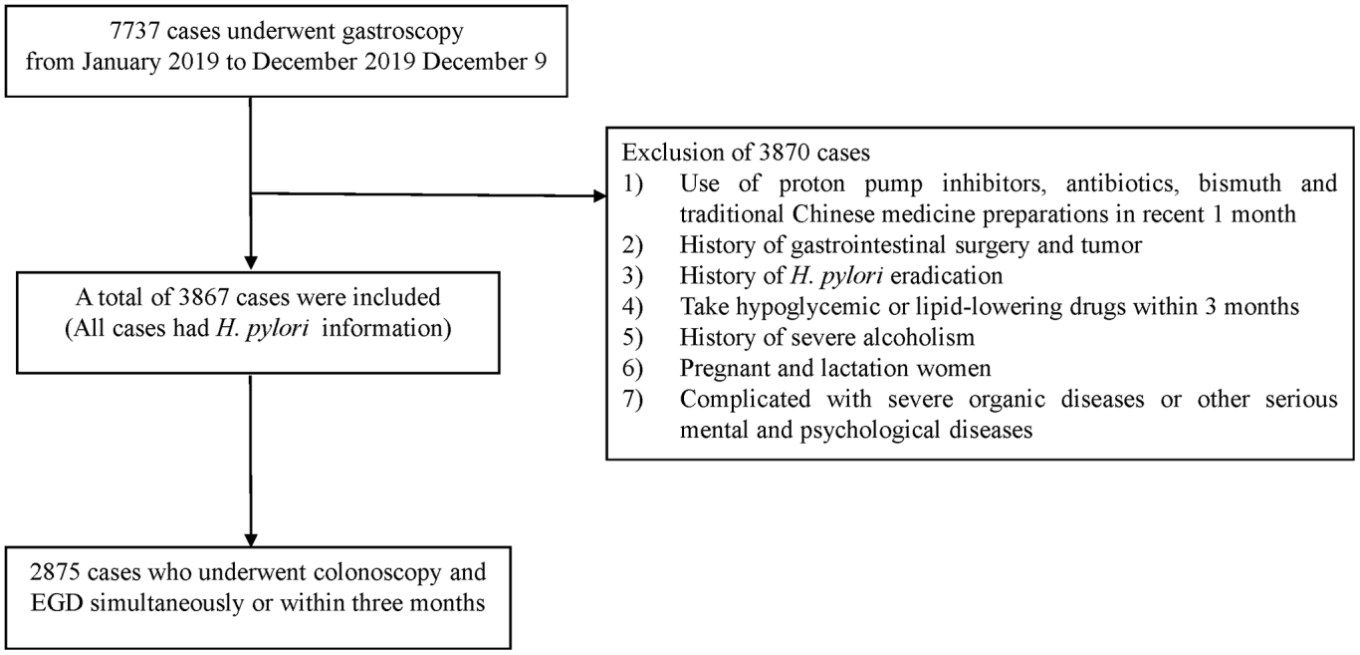
Patient flow chart.

Figure 1 details the results of the logistic regression analysis on the study population. It reveals a significant correlation between age, marital status, and fasting blood-glucose with the risk of *H. pylori* infection. The variables that were significant in univariate analysis (*P* < 0.05) were further examined in multivariate analysis. The findings indicate that age and being married are independent risk factors for *H. pylori* infection (Table 5).

**Table 5:** Multivariate analysis of *H. pylori* infection.

**Variables**	**OR (95% CI)**	***P-*value**
Married	1.435 (1.042–1.977)	0.027^*^
Age	1.007 (1.001–1.013)	0.022^*^
Fasting blood-glucose	1.028 (0.971–1.087)	0.671

## DISCUSSION

The relationship between *H. pylori* infection and upper digestive tract diseases has been extensively studied. *H. pylori* infection could cause histological, chronic and active inflammation, and the inflammatory response will be reduced after *H. pylori* eradication [14]. Therefore, *H. pylori* has been identified as a pathogenic factor that directly causes chronic gastritis and a Class I biological carcinogenic factor in gastric cancer [1]. The underlying mechanisms primarily involve inflammation, reduced acidity, heightened gastrin levels, and a weakened mucosal barrier function [2, 7–11, 15]. In this respect, our study also reached a similar conclusion: the infection rate of *H. pylori* increased significantly in patients with esophageal squamous cell carcinoma, chronic atrophic gastritis, gastric ulcer, duodenal ulcer, gastric MALT lymphoma and gastric cancer, and the differences were statistically significant compared with the non-infected group. But the relationship between *H. pylori* and lower digestive tract diseases is less definitive.

Recently, emerging epidemiologic studies released the connection between *H. pylori* infection and lower digestive tract diseases, particularly colorectal polyp and colorectal cancer. However, these findings remain inconclusive [16–19]. This may be partly attributed to differences in *H. pylori* detection methods, study regions and participant races, as well as small sample sizes. In our study, a relatively large sample size (2875) of patients underwent colonoscopy, among which, 1765 patients were identified by RUC as being infected with *H. pylori*, and the results showed that *H. pylori* infection was positively correlated with the prevalence of colorectal polyps and colorectal cancer.

The association between *H. pylori* infection and colorectal adenoma as well as colorectal cancer may be attributed to the following reasons: prolonged *H. pylori* infection may precipitate chronic atrophic gastritis, consequently impeding gastric acid secretion and disrupting the equilibrium of gastrointestinal flora [20, 21], this disruption can either trigger hypergastrinemia, stimulate the proliferation of colorectal mucosa [22], or ultimately progress to colorectal cancer. Concurrently, *H. pylori* infection can promote the expression of COX-2, whose pivotal role in initiating, progressing, enabling invasion, and facilitating metastasis of colorectal tumors is well-established [23]. Furthermore, studies have documented a heightened positivity rate of *H. pylori* DNA in colon cancer mucosa compared to normal mucosa [24], implying that *H. pylori* may directly provoke intestinal mucosa and induce the occurrence of colorectal lesions.

It is noteworthy that upper and lower gastrointestinal comorbidities are frequently encountered in clinical practice, however, the intricate relationship between these conditions and *H. pylori* infection remains unclear, which is also what we focused on in this study. Our study found that the *H. pylori* infection rate of patients with chronic atrophic gastritis combined with colon cancer or colon polyp was significantly higher than that of the non-comorbidities group; meanwhile, the *H. pylori* infection rate of patients with peptic ulcer or gastric polyp combined with colorectal polyp was also higher than that of the non-comorbidities group. This innovative discovery indicated that gastric mucosa colonization of *H. pylori* not only causes diseases of the upper digestive tract such as chronic atrophic gastritis, but may also affect intestinal health at the same time, this reminds us that when patients are found to have upper digestive tract diseases caused by *H. pylori* infection, it is advisable for them to undergo regular colonoscopy, pathological biopsy, and other monitoring means, especially when they have lower digestive tract symptoms.

Numerous clinical studies have shown that the main risk factors for *H. pylori* infection are related to family living conditions, lifestyle habits, and environmental factors, age and microbiota. Interestingly, our study has uncovered a significant finding: age and marital status may serve as independent risk factors for *H. pylori* infection. As age increases, the immune defense function of the human body also evolves. Simultaneously, the cumulative effect of other risk factors, including adverse family living conditions, smoking and drinking habits, and other unhealthy lifestyle practices, heighten the risk of *H. pylori* infection. The risk of *H. pylori* infection is significantly higher among married individuals compared to singles. This may be attributed to the high *H. pylori* infection rates in China and the oral-to-oral, stomach-to-oral, or fecal-to-oral transmission within families.

This study also has the following limitations. First, our data was derived from a single center in China. Therefore, the generalizability of our findings needs to be validated among other populations. Additionally, in this study, only RUT method was used to detect *H. pylori* infection and all the samples were collected from the gastric antrum, the result of RUT may be affected by many factors, such as the number and site of biopsies taken, the drugs taken by the patient recently (H2 receptor antagonists, proton pump inhibitors, etc.) [25]. Lastly, the long-term impact of *H. pylori* infection duration on digestive tract diseases needs to be further explored.

## CONCLUSION

This study further revealed the *H. pylori* infection was closely related to lower digestive tract diseases, as well as upper and lower gastrointestinal comorbidities. Additionally, being married and advancing age may be independent risk factors for *H. pylori* infection. This is crucial for the management of *H. pylori* infection and the treatment and prevention of gastrointestinal diseases.

## MATERIALS AND METHODS

### Study area

The study was carried out at the Fifth Affiliated Hospital of Sun Yat-sen University, which is situated in Xiangzhou District of Zhuhai City, Guangdong Province, China. The hospital serves the local residents of Xiangzhou District and the surrounding regions, catering to a population of approximately 1.4 million.

### Study design and period

The cross-sectional study design was implemented among patients who underwent gastroscopy during their hospitalization between January 2019 and December 2019.

### Study population

During the study period, 7737 cases underwent gastroscopy. However, 3870 subjects were excluded due to the following reasons: (1) usage of proton pump inhibitors, antibiotics, bismuth, and traditional Chinese medicine preparations within the past month; (2) a history of gastrointestinal surgery and tumor; (3) a history of *H. pylori* eradication; (4) intake of hypoglycemic or lipid-lowering drugs within three months; (5) a history of severe alcoholism; and (6) pregnancy or lactation status; (7) presence of severe organic diseases or other serious mental and psychological diseases. Consequently, 3867 cases were included in the analysis. Additionally, 2875 subjects who had undergone colonoscopy and EGD simultaneously or within three months were included in the study (Figure 1).

### Data collection

The data on social demographics, clinical factors (including sex, nationality, marital status, smoking history, drinking history, living area, age, BMI, glycosylated hemoglobin, fasting blood glucose, total cholesterol, triglyceride), and gastrointestinal endoscopy results were collected from 3867 subjects who met the admission criteria. The endoscopic procedures were carried out by at least one or two experienced gastroscopists. The endoscopic findings were recorded in the endoscopy center database using a descriptive format. The main diagnostic criteria for digestive tract diseases were based on endoscopic and pathological findings. 3867 subjects had available *H. pylori* information detected by rapid urease test (RUT).

### Data analysis and interpretation

The data were entered and analyzed using SPSS version 23.0, produced by SPSS in Chicago, IL, USA. Based on the results of the RUT test, the patients were divided into two groups: the *H. pylori* positive group (2419 cases) and the negative group (1448 cases). For continuous variables, the mean ± standard deviation (SD) was calculated, while categorical variables were reported as frequency (%). The Chi-squared test and Mann-Whitney *U*-test were utilized to compare groups.

A univariate logistic regression analysis was conducted to assess the correlation between *H. pylori* infection and digestive tract diseases. Variables with a *P*-value less than 0.05 were selected for further multivariate analysis. The results are presented as odds ratio (OR) and 95% confidence interval (CI). All two-tailed *P*-values less than 0.05 were considered significant.

## Supplementary Materials

Supplemental File 1
