# Supplementary material for: Relationship between Helicobacter pylori infection and digestive tract diseases and analysis of risk factors: a cross-sectional study based on 3867 Chinese patients
Source: Aging (Albany NY). 2024 Aug 22;16(16):11917–25. doi: 10.18632/aging.206065 (PMC11386919; doi:10.18632/aging.206065)
Supplement: Supplemental File 1 [file aging-16-206065-s001.pdf]

SUPPLEMENTARY TABLES

Supplementary Table 1. *H. pylori* infection status in patients with esophageal squamous cell carcinoma (ESCC) in different locations.

Locations	*H. pylori* infection (+) (*n* = 34)	*H. pylori* infection (−) (*n* = 10)	Total (*n* = 44)	Infection rate	*P*-value
Cervical Esophageal Cancer	5	3	8	62. 50%	
Thoracic Esophageal Cancer	29	6	35	82.86%	$P > 0.05$
Abdominal Esophageal Cancer	0	1	1	0	

Supplementary Table 2. *H. pylori* infection status in patients with colorectal cancer in different locations.

Locations	*H. pylori* infection (+) (*n* = 75)	*H. pylori* infection (−) (*n* = 24)	Total (*n* = 99)	Infection rate	*P*-value
Ascending Colon Cancer	14	7	21	66.67%	
Transverse Colon Cancer	7	2	9	77.78%	
Descending Colon Cancer	5	2	7	71.43%	$P > 0.05$
Sigmoid Colon Cancer	27	8	35	77.14%	
Rectal Cancer	22	5	27	81.48%	
